# CD4 T Helper Cell Subsets and Related Human Immunological Disorders

**DOI:** 10.3390/ijms21218011

**Published:** 2020-10-28

**Authors:** Xiaoliang Zhu, Jinfang Zhu

**Affiliations:** Molecular and Cellular Immunoregulation Section, Laboratory of Immune System Biology, National Institute of Allergy and Infectious Diseases, National Institutes of Health, Bethesda, MD 20892, USA

**Keywords:** Th1, Th2, Th17, Treg, Tfh, ILCs, pathogens, immunological diseases

## Abstract

The immune system plays a critical role in protecting hosts from the invasion of organisms. CD4 T cells, as a key component of the immune system, are central in orchestrating adaptive immune responses. After decades of investigation, five major CD4 T helper cell (Th) subsets have been identified: Th1, Th2, Th17, Treg (T regulatory), and Tfh (follicular T helper) cells. Th1 cells, defined by the expression of lineage cytokine interferon (IFN)-γ and the master transcription factor T-bet, participate in type 1 immune responses to intracellular pathogens such as mycobacterial species and viruses; Th2 cells, defined by the expression of lineage cytokines interleukin (IL)-4/IL-5/IL-13 and the master transcription factor GAΤA3, participate in type 2 immune responses to larger extracellular pathogens such as helminths; Th17 cells, defined by the expression of lineage cytokines IL-17/IL-22 and the master transcription factor RORγt, participate in type 3 immune responses to extracellular pathogens including some bacteria and fungi; Tfh cells, by producing IL-21 and expressing Bcl6, help B cells produce corresponding antibodies; whereas Foxp3-expressing Treg cells, unlike Th1/Th2/Th17/Tfh exerting their effector functions, regulate immune responses to maintain immune cell homeostasis and prevent immunopathology. Interestingly, innate lymphoid cells (ILCs) have been found to mimic the functions of three major effector CD4 T helper subsets (Th1, Th2, and Th17) and thus can also be divided into three major subsets: ILC1s, ILC2s, and ILC3s. In this review, we will discuss the differentiation and functions of each CD4 T helper cell subset in the context of ILCs and human diseases associated with the dysregulation of these lymphocyte subsets particularly caused by monogenic mutations.

## 1. Introduction

To defend countless pathogen invasions, mammals have developed and evolved a rigorous and precise immune system, which is built upon various kinds of cells, cytokines, chemokines, antibodies et al. [1,2]. CD4 T helper cells, as a key component of this system, play a critical role in defending infections throughout life by coordinating the actions of other immune cells as well as non-immune cells. CD4 T cells originate from common lymphoid progenitors in the bone marrow, develop in the thymus, and execute their functions mainly in peripheral tissues and various lymphoid organs [3]. The developmental process is precisely regulated so that CD4 T cells may efficiently recognize a very broad range of foreign antigens without eliciting auto-reactivity to self-antigens. For executing their effector functions, naïve CD4 T cells first need to be activated when receiving pathogen signals delivered by antigen-presenting cells (APCs), such as dendritic cells (DCs), and then differentiate into distinct T effector (Teff) populations based on the nature of infections. Effector CD4 T cells provide host defensive mechanisms against pathogens through the production of effector cytokines, which activate other immune cells such as macrophages and CD8 T cells in killing infected cells and help B cells in producing antibodies to mediate humoral immune responses [4,5,6].

**Figure 1:**
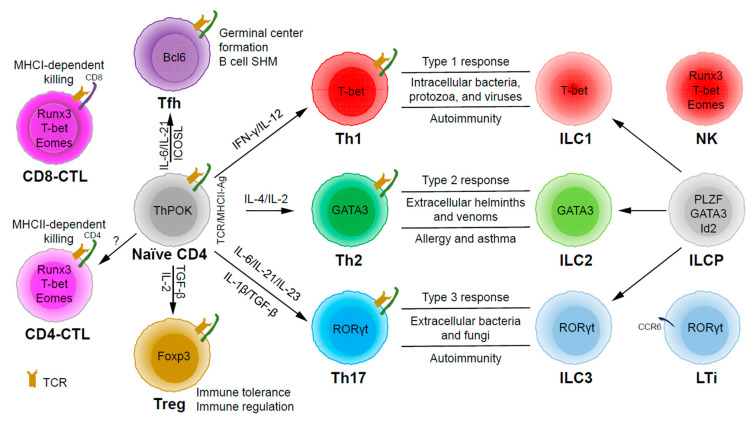
The development and functions of CD4 T helper (Th) and innate lymphoid cell (ILC) subsets. Naïve CD4 T cells can differentiate into Th1, Th2, Th17, Tfh (follicular T helper), and Treg (T regulatory) subsets upon T cell receptor (TCR) activation in different cytokine milieu. T cells may also become cytotoxic CD4 T cells (CD4-CTLs) that kill their target cells in major histocompatibility complex class II (MHCII)-restricted manner. ILC1, ILC2, and ILC3 subsets are the innate counterparts of Th1, Th2, and Th17 cells and they are involved in type 1, type 2, and type 3 immune responses, respectively. ILC1, ILC2, and ILC3 subsets develop from ILC progenitors (ILCPs), which are distinct from progenitors that give rise to natural killer (NK) cells and lymphoid tissue inducers (LTis).

Based on the expression of signature cytokines and lineage-specific master transcription factors, CD4 T helper (Th) cells are typically categorized into five major subsets (Figure 1): Th1 (interferon (IFN)-γ and T-bet), Th2 (interleukin (IL)-4/IL-5/IL-13 and GATA3), Th17 (IL-17/IL-22 and RORγt), Tfh (IL-21 and Bcl6) and Treg (T regulatory) (IL-10/transforming growth factor (TGF)-β/IL-35 and Foxp3) [7]. Some labs have also described some other subsets, such as Th3 (TGF-β) [8], Tr1 (IL-10) [9], Th9 (IL-9) [10,11], and Th22 (IL-22) [12]. Since there are still controversial issues in defining them as unique lineage subsets (particularly because the lack of a master regulator associated with each of these “lineages”), we will not further discuss these CD4 T cell subsets in this review.

Through thymic selection, CD4/CD8 double positive T cells develop into either mature CD4 (helper) or CD8 (cytotoxic) T cells, which recognize major histocompatibility complex (MHC) class II (MHCII)/peptide and MHCI/peptide complexes, respectively (Figure 1). However, some mature CD4 T cells, cytotoxic CD4 T cells (CD4-CTLs or Th-CTLs), may act similarly as cytotoxic CD8 T cells in killing their target cells through the secretion of granzyme B and perforin but in an MHCII-restricted manner [13,14,15]. The transcriptional factor Eomes (a T-box transcription factor critical for the development of functional natural killer (NK) and CD8 T cells), T-bet, and Blimp1 are critical for inducing the expression of cytotoxicity-associated genes in CD4-CTLs [16,17,18]. Similar to CD8 T cells, CD4-CTLs display increased Runx3 but reduced ThPOK expression [19,20,21,22]. Furthermore, opposite to Tfh cells, CD4-CTLs express low levels of Bcl6 but high levels of Blimp-1 [23]. Two important markers for CD4-CTLs are CRTAM [24] and NKG2C/E [25]. It has been shown that the cytotoxicity executed through CD4-CTLs becomes effective when CD8-CTL activity is impaired during infections in association with virus escape strategies [14,15]. Interestingly, more CD4-CTLs have been identified in severe COVID-19 patients than in mild symptomatic patients [26], suggesting a possible inefficient CD8 response in viral control in patients with severe disease, which is an important question that needs to be tested in the future. In addition to protecting the host from viral infections, CD4-CTLs are also involved in anti-tumor responses as well as in various autoimmune conditions [19,27,28]. Strikingly, CD4-CTLs are increased in people of exceptional longevity [29]. The development and functions of CD4-CTLs have been recently reviewed [15]. In consideration of the scope of this review, we will not further discuss this interesting population below in great detail.

Among the CD4 T subsets possessing helper functions (Figure 1), Th1 cells induce type 1 responses to protect against intracellular pathogens such as bacteria, viruses, and protozoa by activating type 1 macrophages (M1) [5]. Th2 cells are involved in type 2 responses to fight infections by parasites such as helminths by activating type 2 microphages (M2), as well as the recruitment of eosinophils, basophils, and mast cells to the sites of infections [30]. Th17 cells mediate type 3 responses for the clearance of extracellular bacteria and fungi by orchestrating sustained neutrophil recruitment and an induction of antimicrobial peptide production by epithelial cells of barrier tissues such as intestines, lungs, and skin [31]. Tfh cells are found to be important in helping B cells make antibody responses by promoting germinal center formation, affinity maturation, and possibly immunoglobulin class switch recombination [32], although Tfh-independent antibody responses have also been reported [33]. Treg cells are critical for the preservation of immune tolerance and involved in preventing autoimmune diseases [34,35].

Innate lymphoid cells (ILCs) [7,36,37] that do not express antigen receptors have been recently identified as the counterparts of T helper subsets within the innate arm of the immune system. While progenitors of ILCs are found both in bone marrow and tissue, mature ILCs are largely tissue-resident [38]. Therefore, ILCs can act earlier and more quickly than CD4 T cells during an immune response and thus provide the first line of protection against pathogens through a rapid and local secretion of effector cytokines, which were previously thought to be largely produced by CD4 T helper cells. Similar to the classification of CD4 T effector subsets into three major subsets, Th1/Th2/Th17 cells, ILCs can also be categorized into three major groups (Figure 1), which produce similar effector cytokines and lineage-defining transcription factors. Thus, group 1 ILCs (ILC1s) secret IFN-γ and express T-bet; group 2 ILCs (ILC2s) secret IL-5 and IL-13, and express GATA3; group 3 ILCs (ILC3s) secret IL-17 and IL-22, and express RORγt [7]. Functionally, group 1 ILCs, similar to Th1 cells, may participate in defending intracellular pathogens, such as bacteria, viruses, and protozoa; group 2 ILCs, just as Th2 cells, respond to large extracellular pathogens, such as parasites and allergens; group 3 ILCs, like Th17 cells, combat extracellular microbes, such as bacteria and fungi [37]. ILCs, similar to Th cells, are also involved in many inflammatory diseases including autoimmunity, allergy, and asthma [39]. It is worth pointing out that within the RORγt-expressing ILCs, there is a lymphoid tissue inducer (LTi) population, and they are involved in the formation of lymphoid structures as well as in host defense [40]. Recently, ILCs were also found to be involved in non-immunological functions including tissue repair, metabolism regulation, and immune-neuronal interactions [36].

A proper and effective response to a particular microorganism invasion through the activation and differentiation of T helper subsets and ILCs protects host from that pathogen without causing excessive inflammation that damages host tissues. However, when an immune response is out of balance, either too weak or too strong, it can cause chronic infection or inflammatory diseases [5,6,32,41,42]. Inappropriate immune responses to harmless environmental agents or self-antigens may also lead to inflammation, allergy, and autoimmunity, such as inflammatory bowel disease (IBD). Immunodeficiency with reduced ILC and CD4 T cell numbers and/or diminished ILC and CD4 T cell functions can result in insufficient immune response, which may cause chronic or repeated infections. In fact, in human immunodeficiency virus (HIV)-infected patients, the CD4 T cell number is drastically reduced, which is the main cause of acquired immunodeficiency syndrome (AIDS). X-linked severe combined immune deficiency (SCID) patients have mutations in the *IL2RG* gene [43], which results in a lack of T cells and ILCs; these boys suffer from severe infections of bacteria, viruses, and fungi, and they do not survive beyond infancy without treatment. In this review, we will discuss the differentiation and functions of major T helper subsets and their involvement in host defense and diseases in the context of ILCs.

## 2. Th1 Cells and Related Diseases

### 2.1. Th1 Cells

The Th1/Th2 dichotomy was first proposed by Robert Coffman and Tim Mosmann in 1986 when they reported that CD4 T helper cell clones from mice can be divided into two distinct types based on their cytokine production profile [44]. Since then, the definition of a unique lineage has expanded to the expression of lineage-specific master transcription factors, cell surface markers, as well as transcriptomes and epigenomes, which is reflected by different epigenetic modifications to a certain degree [5].

Upon TCR activation in a particular cytokine milieu, naïve CD4 T cells can differentiate into Th1 cells. IL-12 secreted by APCs activates the transcription factor STAT4, and IFN-γ produced by NK cells and/or T cells themselves activates another transcription factor, STAT1; both STAT1 and STAT4 activation are capable of inducing the expression of the Th1-inducing master transcription factor T-bet [45,46]. T-bet, by cooperating with Hlx [47], Runx3 [48,49], Ets-1 [50], and Bhlhe40 [51], promotes IFN-γ production. While T-bet together with Runx3 may directly repress IL-4 transcription, T-bet also inhibits the expression of other master transcription factors including GATA3 and RORγt [7,45,52], thereby antagonizing Th2 and Th17 cell differentiation.

While Th1 cells can directly differentiate from naïve CD4 T cells, they can also derive from other T helper CD4 subsets, including Th17, Treg, and Tfh cells as a result of CD4 T cell plasticity [5,53,54]. Differentiated Th1 cells are capable of producing Th1 signature cytokine IFN-γ, which activates and/or stimulates other immune cells, including CD8 T cells, ILC1s, macrophages, and B cells during the process of eliminating pathogens [6]. An important chemokine receptor expressed by Th1 cells is CXCR3, which plays an important role in Th1 cell migration toward the inflammation sites with pathogen invasion, and it is also widely used for the identification of human Th1 cells [55].

In addition to Th1 cells, three other kinds of lymphocytes (ILC1s, CD8 T cells, and NK cells) are also involved in type 1 immunity [6]. ILC1s, probably by producing IFN-γ, may participate in immune responses to the infection of protozoa and viruses [56,57]. However, the relative importance of IFN-γ production by ILC1s or NK cells during infection remains unclear partly because of the lack of reliable ILC1-deficient models. It has been recently reported that ILC1s are essential for limiting early viral replication, which cannot be compensated by NK cell-mediated anti-viral effects in response to mouse cytomegalovirus infection of hepatic cells [57]. However, the transcription factor Zfp683 (Hobbit), which appeared to be specifically required for ILC1 development, may also have important functions in other immune cells including NK, CD8 T, and Th1 cells. Whether IFN-γ produced by ILC1s plays a role in promoting Th1 cell differentiation is currently unknown.

Whole genome analyses of Th1 cells generated both in vitro and in vivo, including RNA-Seq for assessing gene expression, ChIP-Seq of histone modifications, and transcription factor binding as well as ATAC-Seq (and/or DNase-Seq) for analyzing epigenetic status of specific gene loci, have not only confirmed the previously known Th1-related genes but also identified many more Th1-specific genes, which may help understand the mechanisms of Th1 cell differentiation as well as the heterogeneity and functions of Th1 cells after their differentiation [58,59,60,61].

### 2.2. Th1-Related Diseases

In addition to an essential protective effect in host defense, Th1 cells also play important roles in (or are associated with) different types of autoimmune diseases, such as rheumatoid arthritis, multiple sclerosis, Hashimoto thyroiditis, insulin-dependent diabetes mellitus, autoimmune gastritis, and other chronic inflammatory disorders, including inflammatory bowel diseases, atherosclerosis, and sarcoidosis [62]. Since Th1 cells are responsible for orchestrating type 1 immunity against intracellular pathogens, deficiencies in the generation and functions of Th1 cells may result in chronic and/or recurrent infections. However, no monogenic mutations only related to Th1 cells have been found [5]; this may reflect a complex connection and interaction network within our immune system.

Serving as the master regulator for Th1 cell differentiation and IFN-γ production, T-bet has recently been reported to be deficient in some patients with mycobacterial disease [63]; these patients have reduced IFN-γ production by most of the innate-like lymphocytes including NK, invariant NKT (iNKT), mucosal-associated invariant T (MAIT), and Vδ2^+^ γδ T cells. Interestingly, while non-mycobacterial reactive classic Th1 cells produced less IFN-γ, normal IFN-γ response was detected in mycobacterial reactive non-classic CCR6^+^ Th1 (Th1*) cells, indicating that T-bet-independent Th1-related response may be elicited and/or preserved in a situation where innate and classic adaptive IFN-γ response is inadequate. Μutations in many other genes that are related to Th1 cell differentiation and/or functions, such as *IFNGR1, IFNGR2, STAT1, IL12RB1, IL12B* and *NEMO*, have also been found in patients [64]. The vast majority of these abnormalities result in susceptibility to infections with mycobacterial species.

Since there is a similarity in the development and regulation of ILC1s, NK, and Th1 cells, some gene mutations that affect Th1 cell differentiation may also have an impact on the generation and functions of other type 1 lymphocytes. Indeed, T-bet deficiency has a broad impact on multiple lymphoid lineages in either development/maturation or IFN-γ production [63]. Th1 cells may also be involved in tumor immunology. Interestingly, the epigenetic silencing of Th1-type chemokines has been reported as a novel immune-evasion mechanism of tumors [65]; this indicates that a selective epigenetic reprogramming may enhance the clinical efficacy of cancer therapy.

## 3. Th2 Cells and Related Diseases

### 3.1. Th2 Cells

Th2 cells are defined as IL-4-producing T cells when the Th1/Th2 model was proposed. Th2 cells are mainly involved in host defense against large extracellular pathogens, such as helminths. Naïve CD4 T cells after receiving signals from DCs may differentiate into Th2 cells after their activation [30]. IL-2 and IL-4 are two important cytokines during Th2 cell differentiation, particularly in vitro. IL-2 and IL-4 activate STAT5 and STAT6, respectively, and the latter induce the expression of the Th2 master transcription factor GATA3 in activated CD4 T cells [5]. IL-4-independent Th2 cell differentiation may occur in vivo [66]; however, GATA3 is absolutely required for Th2 cell differentiation and functions both in vitro and in vivo [67,68]. GATA3 not only plays an essential role in inducing Th2 cell differentiation, it also represses the expression of other lineage transcription factors, such as T-bet and RORγt [58,69,70]. In addition to GATA3, other transcription factors including c-Maf [71], STAT3 [72], and Notch/CSL [73] may also regulate Th2 cell differentiation and functions.

Differentiated Th2 cells secret lineage cytokines including IL-4, IL-5, and IL-13. During a classic type 2 immune response, IL-4 promotes B cell antibody class switching to immunoglobin E (IgE), IL-5 recruits eosinophilia to the inflammation sites, and IL-13 promotes mucus production and goblet cell hyperplasia [30]. These cytokines can also be produced by ILC2s (although IL-4 production by ILC2s is somewhat limited), and they work together to eliminate invading pathogens [74]. Th2 cells and ILC2s also express chemokine receptors on the cell surface, including CCR3, CCR4, and CCR8, to receive migration signals from ligands (CCL11 for CCR3, CCL17 and CCL22 for CCR4, and CCL1 for CCR8) secreted by epithelial cells [75,76,77]. In addition to recruiting Th2 cells and ILC2s, these chemokines can also recruit other cells, such as DCs, eosinophils, basophils, and mast cells to the damaged epithelial sites caused by helminth infection or protease allergens [30,78]. Human Th2 cells also express CRTh2, which serves as a reliable marker for identification of human Th2 cells [79], although its expression in mouse Th2 cells has not been clearly demonstrated. Instead, T1/ST2 (an IL-33 receptor subunit) has been widely used to identify Th2 cells and ILC2s in mice [80,81].

**Figure 2:**
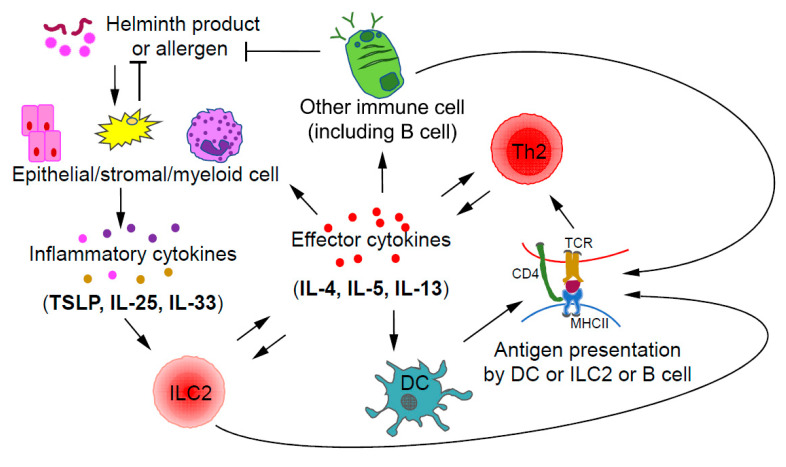
Crosstalk between ILC2s and Th2 cells during type 2 immune responses. Upon stimulation by helminth products or allergens, epithelial and/or stromal cells may produce inflammatory cytokines including IL-33, IL-25, and TSLP, which are capable of activating ILC2s. By producing type 2 effector cytokines, such as IL-4 and IL-13, ILC2s may promote the migration of dendritic cells (DCs) into draining lymph nodes to induce Th2 cell differentiation. Some ILC2s also express MHCII, which allows them to directly interact with Th2 cells through antigen presentation. Thus, ILC2s could promote the further differentiation of early Th2 cells; on the other hand, Th2 cells may expand ILC2s through IL-2 production. Effector cytokines including IL-4 and IL-13 produced by Th2 cells, may also indirectly induce the expansion of ILC2s through stimulating epithelial cells to produce ILC2-activating inflammatory cytokines such as IL-25 and TSLP.

Collaboration and interactions between the ILCs and Th cells of the same class during immune responses have been reported with the relationship between ILC2s and Th2 cells being the most certain one [30]. During a type 2 immune response, ILC2s and Th2 cells may crosstalk to each other through direct and indirect mechanisms (Figure 2). After activation by inflammatory cytokines, such as IL-33, IL-25, and TSLP, ILC2s, by secreting IL-13, may promote the migration of DCs to draining lymph nodes to induce Th2 cell differentiation [82]. Interestingly, some ILC2s also express MHCII, through which they may directly present antigens to Th2 cells [83]. On the other hand, Th2 cells by secreting IL-2 may expand ILC2s. Furthermore, by secreting IL-4 and IL-13, Th2 cells can induce/enhance the production of ILC2-activating inflammatory cytokines by epithelial cells, forming a positive feedback loop at the cellular level [84,85]. Since ILC2s and Th2 cells have similar effector functions during type 2 immune responses and both can be activated by IL-33, the relative abundance of one population over the other at a particular stage may determine the relative importance of these cells at that moment [86].

The heterogeneity of Th2 cells, particularly in vivo, has been documented [87]. In fact, even GATA3^+^T-bet^+^ and GATA3^+^RORγt^+^ CD4 T cells have been found during type 2 immune responses [88,89,90]. Such an appearance of mixed phenotype cells is possibly due to CD4 T cell plasticity [91]. Whole genome analyses of Th2 cells have also identified a collection of Th2-specific genes, many of which are shared by their expression in ILC2s. The investigation of these molecules may help further understand the mechanisms of Th2 cell differentiation and ILC2 development as well as their unique and shared functions during helminth infection and allergic responses [58,70,92,93].

### 3.2. Th2 Related Diseases

Th2 cells play a central role in type 2 immune responses, and together with ILC2s, Th2 cells defend against large parasites such as helminths [30]. On the other hand, the dysregulation of type 2 immune response often results in pathologic responses [30,94]. In addition to some well-known large parasites such as helminths, other stimulus derived from viruses, bacteria, and other nonmicrobial products including food allergens [95], venoms [96], interior allergens (such as house dust mites, HDM) [97], pollen allergens [98] and vaccine adjuvants (Alum, MF59) [99], may also cause allergic inflammation including asthma, atopic dermatitis, rhinitis, food allergies, and eczema. GATA3, as the master transcription factor for Th2 cells, plays an essential role in Th2 cell differentiation and functions [68]. In mice, *Gata3* germline deficiency results in embryonic lethality [100]. It is probably true in humans as well. Therefore, only *GATA3* heterozygous mutations that cause a haploinsufficiency of GATA3 in the hypoparathyroidism, deafness, and renal dysplasia (HDR) patients have been reported [101]. Th2 cell differentiation was reported to be partially defective in these patients’ T cells; however, they do not show any major immunologic abnormalities [102]. Consistently, another report showed that the differentiation of HDR patient T cells into Th2 cells is normal [103]. However, since ILC2 development is sensitive to the dose of GATA3 expression [104], whether the HDR patients have reduced ILC2 numbers and thus reduced ILC2-mediated immune responses remains to be tested.

RAG1 or RAG2 hypomorphic mutations are responsible for Omenn syndrome, which is an autosomal recessive disease of neonates characterized by erythroderma, chronic diarrhea, hepatosplenogaly, and lymphadenopathy [105,106]. Elevated numbers of Th2 cells, an overproduction of IL-4 and IL-5, and elevated serum IgE as well as eosinophilia were found in some of these patients. This is possibly because a partial RAG deficiency results in a limited T cell repertoire, which may lead to a Th2-biased disease. Consistent with this idea, transferring low numbers of mouse T cells (thus a reduced T cell repertoire) into a lymphopenic host also causes Th2-mediated diseases [107].

## 4. Th17 Cells and Related Diseases

### 4.1. Th17 Cells

Th17 cells are important for type 3 immune responses in defending extracellular bacteria and fungi invasion; they are also found to be involved in many autoimmune diseases, such as multiple sclerosis, because of their pathogenic effects [108]. Several key reports on the role of IL-23 versus IL-12 in autoimmunity in 2003 [109,110,111] lead to the discovery of Th17 cells in 2005 [112,113,114]. Although the discovery of Th17 cells is much later than the identification of Th1 and Th2 cells, research on this unique CD4 subset progresses rapidly. Interestingly, more regulators have been identified for Th17 cell differentiation than for Th1/Th2 cell differentiation [115,116]. Products of extracellular bacteria and fungi can activate antigen-presenting cells to produce pro-inflammatory cytokines IL-1β, IL-6, and IL-23, which in turn direct naïve CD4 T cells to differentiate into Th17 cells [6]. IL-6 and IL-23 activate STAT3 to induce the expression of Th17 master transcription factor RORγt [117,118]. In addition, TCR signaling can induce RORγt expression through the NFAT/NF-κB/AP-1 pathway [5]. RORγt, by collaborating with other transcription factors such as IRF4, BATF, and Runx1, promotes the expression of Th17 lineage-specific genes [5,119]. IL-6 and IL-21 together with TGF-β are involved in Th17 cell differentiation in vitro [5], although the TGF-β-independent pathway for Th17 cell differentiation has also been reported [120]. Differentiated Th17 cells are capable of secreting effector cytokines, such as IL-17A, IL-17F, and IL-22, to activate nonimmune and immune cells to produce matrix metallopeptidases (MMPs), nitric oxide (NO), cytokines, and antimicrobial peptides to clear extracellular pathogens [6,112,114,121,122]. In addition, CXCL8 and G-CSF produced by Th17 cells and other nonimmune cells recruit neutrophils to the inflammation sites to kill pathogens [123]. Th17 cells express a signature chemokine receptor CCR6 (inflamed tissues homing receptor and essential for homeostasis) [124], which is also considered as a unique marker in identifying human Th17 cells. In combination with CXCR3, CCR6 has been used to distinguish Th17 cells (CXCR3^-^CCR6^+^) from Th1 cells (CXCR3^+^CCR6^-^) and Th1* cells (CXCR3^+^CCR6^+^) [125,126].

ILC3s are also involved in type 3 immune responses [37]. IL-22 production by ILC3s is important for host defense against extracellular bacteria [127]. IL-22 induces the production of antimicrobial peptides by epithelial cells. In addition, ILC3-derived IL-22 may induce the expression of serum amyloid proteins, which promote IL-17 production by Th17 cells [128]. On the other hand, Th17 cells may silence IL-22 production by ILC3s through controlling commensal bacteria; the sequential activation of ILC3s and Th17 cells has been noted during normal development, while steady-state commensalism is being established in the host, and in the absence of Th17 cell differentiation, ILC3s remain constitutively active, leading to an impaired lipid metabolism in the gut [129]. Therefore, there is a crosstalk between ILC3s and Th17 cells in maintaining gut homeostasis.

### 4.2. CD4 T Cell Plasticity

Although cell plasticity has been found in almost all CD4 T cell subsets, Th17 cells are the most flexible in altering their phenotypes [53,130]. Now, many current studies focus on Th17 cells’ dichotomous nature (protective and pathogenic effects), plasticity, and its application in therapy [31]. Various important extracellular and intracellular regulators of Th17 cells have already been found [119]. There are several regulators, such as IL-23 [131], CD5L [132], and REV-ERBα [133], that have been found recently to be involved in regulating Th17 dual functions. Although the detailed mechanism behind their dichotomous nature is still unclear, it may have something to do with Th17 cell plasticity being influenced by the environment. Indeed, several environmental factors, such as sodium chloride [134], microbiota [135,136,137], and angiogenesis [138], may regulate the generation of pathogenic Th17 cells. In terms of cell plasticity, Th17 cells were reported to be unstable both in vitro and in vivo, and they may become Th1-like cells [53,139]. Glutamine metabolism was found to be important in regulating Th17 versus Th1 cell differentiation [140,141]. CK2 was reported to control Th17 and Treg cell differentiation through inhibiting FoxO1 [142]. Recently, it has been found that metabolic heterogeneity may underlie reciprocal fates of Th17 cell stemness and plasticity [143]. In a mouse model of autoimmune disease, it was found that Th17 cells are functionally and metabolically heterogeneous and contain two different subsets: one subset with stemness-associated features but lower anabolic metabolism, and another subset with higher metabolic activity that supports trans-differentiation into Th1-like cells. This study was also supported by another study, which reported a metabolism difference related with the degree of Th17 cell plasticity [144]. Since IL-12 and IL-23 are responsible for converting Th17 cells to Th1-like cells [52,53,145], the relationship between the production of IL-12/IL-23 and metabolic regulation requires further investigation.

### 4.3. Th17-Related Diseases

As discussed above, Th17 cells contribute to immunity against extracellular bacteria and fungi and the pathogenesis of multiple chronic inflammatory diseases [5,66]. Patients with mutations in the *RORC* (which encodes RORγt) gene suffer from chronic candidiasis consistent with a lack of Th17 cells in these patients [146]. Interestingly, these patients are also susceptible to mycobacterium infection with reduced IFNγ response. Whether this is due to a defect in thymic development in these patients (RORγt also plays an important role in T cell development) or due to a blockage of the development of Th1 cells from a transient Th17 stage requires further investigation. *STAT3*, as the gene encoding a key transcription factor for Th17 cell differentiation, was found to be mutated in many patients with hyper-IgE syndrome, which is a primary immunodeficiency disorder [147,148,149,150]. These patients have heightened susceptibility to be infected by bacteria, such as *Staphylococcus aureus*, *Streptococcus pneumoniae, Candida albicans*, and other fungi due to impaired Th17 cells. It was also found that mutations in *DOCK8* may result in hyper-IgE syndrome [151,152], and patients with this form of mutation have a deficiency in Th17 cells. It was reported that mutations that take place in the Src homology 2 (SH2)-/tail segment (TS)-/transactivation (TA)-/DNA binding-domain of STAT1 result in loss-of-function in Th1, and patients with this form of mutation display increased susceptibility to mycobacteria and viruses [153,154]. However, mutations that take place in the coiled-coil domain of STAT1 cause enhanced Th1 but reduced Th17 cell-mediated responses; patients with this form of mutation suffered from chronic mucocutaneous candidiasis [155,156]. Mechanistically, this mutation within the coiled-coil domain of STAT1 reduces the dephosphorylation of activated STAT1 and thus increases the level of phosphorylated STAT1 in the nucleus [155]. As a result, the dominance of activated STAT1 causes an overproduction of IFN-α/β, IFN-γ, and IL-27, which induce the STAT1-mediated inhibition of Th17 cell differentiation. It has also been reported that two different genetic etiologies of chronic mucocutaneous candidiasis diseases are associated with autosomal recessive deficiency in the cytokine receptor IL-17RA and autosomal dominant deficiency in IL-17F, respectively [157]. In addition, due to the important role of IL-1β during Th17 cell differentiation and/or stabilization [117,158], mutations in IL-1β were found to cause several autoinflammatory disorders [159].

Given that Th17 cells are closely associated with multiple inflammatory and autoimmune disorders, targeting these cells or their effector cytokines is considered to be an effective way for disease treatment. For example, antibodies against IL-12p40 (the common subunit of IL-12 and IL-23) have been successfully used in treating psoriasis [160,161]. Similarly, anti-IL-17A/F, anti-IL-17RA, and RORγt inhibitors have been used in treating different types of autoimmune diseases [162,163].

## 5. Treg Cells and Related Diseases

### 5.1. Treg Cells

It has been long speculated that a specialized population of lymphocytes in mammals can perform an immune regulatory function. However, no one was able to provide strong evidence for the existence of these cells until a breakthrough report in 1995, in which Sakaguchi and his colleagues described that a subset of CD4 T cells marked by CD25 expression are essential for the maintenance of self-tolerance [164]. Another groundbreaking discovery came in 2003, when three groups independently identified Foxp3 as the master transcription factor for Treg cells [165,166,167].

Regulatory T cells can develop in the thymus during negative selection, and these Treg cells are called thymic-derived Treg (tTreg) cells [168]. tTreg cells, by recognizing self-antigens, mainly control self-reactive effector T cells that escaped negative selection to maintain immune tolerance [169]. Treg cells can also be induced in vitro from naïve CD4 T cells, and they are now called inducible Treg (iTreg) cells [170,171,172,173,174]. In contrast, Treg cells differentiated from naïve CD4 T cells in vivo outside of the thymus are now named as peripherally induced Treg (pTreg) cells. The differentiation of pTreg cells, especially in the gut [175], is promoted by retinoic acid in the environment and metabolites produced by microbiota [176,177]. Compared with tTreg cells, pTreg cells not only recognize self-antigens, but also recognize non-self-antigens including food antigens and innocuous commensal microbiota-derived antigens; therefore, they are important for the maintenance of mucosal tolerance [178]. Several markers, including Helios and neuropilin (Nrp), have been proposed to distinguish pTreg cells from tTreg cells [179,180]; however, these molecules can also be expressed by some pTreg cells, indicating that they may simply mark more differentiated Treg cells. Similarly, it has been proposed that tTreg cells are more stable than pTreg and iTreg cells, but this could also be explained by the more differentiated status of tTreg cells compared to other Treg cells [181,182]. In the gut, RORγt-expressing Treg cells may represent pTreg cells, whereas GATA3-expressing Treg cells could be largely tTreg cells [183,184].

Cytokines, especially IL-2 and TGF-β, play essential roles during the development and differentiation of Treg cells. IL-2 and TGF-β promote Foxp3 expression by activating STAT5 and SMAD2/3, respectively [185,186,187]. TCR signaling is also important and it induces Foxp3 expression through the activation of the NF-AT/NF-κB/AP-1 pathways [170].

In addition to Foxp3, many other transcription factors, such as Runx1 [188], Nrfa2 [189], Foxo family members [190], SATB1 [191], and Helios [179,192], can regulate the development and functions of Treg cells. Multiple mechanisms through which Treg cells exert their regulatory functions have been proposed: by secreting regulatory cytokines (such as IL-10, TGFβ, and IL-35) [193]; by consuming IL-2, which is important for effective CD4 T cell survival and proliferation [194,195]; by expressing cell surface receptors that transmit negative signals (such as CTLA-4, CD39, CD73) [34,196]; by expressing perforin and granzyme B to directly kill APCs [197]; and by inducing trogocytosis to strip off the antigen-MHCII complexes from APCs in an antigen-specific manner [198].

### 5.2. Treg Related Diseases

Since Treg cells are essential for the maintenance of immunological tolerance and Foxp3 is the master transcription factor for Treg cells, mutations in the *Foxp3* gene in mice or in the *FOXP3* gene in humans lead to severe autoimmune diseases and death [199,200,201]. Since the *FOXP3* is located in the X chromosome, boys carrying a deleterious *FOXP3* mutation will develop immunodysregulation polyendocrinopathy enteropathy X-linked (IPEX) syndrome. The syndrome of IPEX often includes insulin-dependent diabetes, elevated serum IgE with aspects of both eczema and psoriasis, and some other autoimmune symptoms include hypothyroidism, anemia, thrombocytopenia, and neutropenia. Hemizygous male mice with a mutant *Foxp3* (*scurfy* mice) develop lethal multi-organ autoimmunity and die 16–25 days after birth. Some *FOXP3* mutations cause less severe diseases. For example, patients with a point mutation in the domain swap interface of FOXP3 protein develop a Th2-related disease [202]. In addition to Foxp3 mutations, deficiency in other regulators of Treg-suppressive functions, such as TRAF3 [203], CD28 [204], Id2/Id3 [205], Ubc13 [206], Ndfip1 [207], NF-κB p65 [208], Helios [209], Th-POK/LRF [210], EZH2 [211], BACH2 [212,213], SATB1 [214], IRF-4 and Blimp-1 [215], and LKB1 [216], can also result in severe autoimmune diseases, although most of these studies were carried out in mice, and their relevance in human diseases remains to be tested.

## 6. Tfh Cells and Related Diseases

### 6.1. Tfh Cells

Tfh cells were noticed about twenty years ago but only well accepted as a distinct lineage of CD4 T cells required for the formation of germinal centers (GCs) when its master transcription factor Bcl6 was identified [217,218,219]. Tfh cells are mainly found in the B cell follicles in lymph nodes and spleen, so they may help B cells in antibody production and affinity maturation [220]. How important Tfh cells are for antibody class switching remains to be tested, since antibody class switching happens largely outside of GCs [221]. Similar to the differentiation of other CD4 T cell subsets, Tfh cell differentiation from naïve CD4 T cells is also tightly regulated by TCR signaling and cytokine milieu [32]. Possibly because of an essential role of the interaction between antigen-specific B cells and pre-Tfh cells during Tfh cell development, it is difficult to generate Tfh cells in vitro [222,223]. Nevertheless, IL-6 and IL-21 by activating STAT3 were found to induce Tfh cell differentiation. IL-6 together with TCR signaling is involved in the initiation of Tfh cell differentiation by inducing an early wave of Bcl6 expression [218,224]. IL-21 was also reported to be involved in Tfh cell differentiation [225]. Unlike IL-6 and IL-21, IL-2 [219,226] plays a negative role during Tfh cell differentiation by promoting the expression of Blimp-1, which inhibits Bcl6 expression. By producing cytokines or direct cell-cell contact, both DCs and B cells play an essential role in promoting Tfh differentiation and migration [227]. After the activation of naïve CD4 T cells in the T cell zone by DCs, these activated T cells may migrate to the T-B border through upregulating CXCR5 and downregulating CCR7 expression; with further upregulation of CXCR5 expression after in contact with B cells, Tfh cells continue to migrate into GC to become GC Tfh cells [228]. In addition to CXCR5, CCR7, PSGL1, and S1P receptors that are involved in cell migration, SLAM family receptors and integrins are also involved in regulating Tfh cell functions [32]. In addition, other regulators, including ICOSL [222], Ascl2 [229], Itch [230], VHL [231], IL-37 [232], HIF-1α [233], COX-1 [234], Th-POK [235], and Tox2 [236] are also found to be important for Tfh cell differentiation and functions. Tfh cells provide help to GC B cells in two aspects: one is to promote GC B cell maturation, and the other is to promote antibody production. CD40L expressed on Tfh cells is required for inducing the formation of high-affinity plasma cells and memory B cells [237,238]. Signature cytokine IL-21 secreted by Tfh cells may control the maintenance and optimal affinity maturation of GC responses [220,239,240].

### 6.2. Tfh Subsets

Although the main cytokine produced by Tfh cells is IL-21, it was found that Tfh cells can also secret cytokines that are produced by Th1, Th2, and Th17 cells; therefore, Tfh cells can be classified into Tfh1, Tfh2, and Tfh17 cell subsets [241]. Recently, it has been reported that a new subset of Tfh, which is referred to as Tfh13 cells, have an unusual cytokine profile (IL-13^hi^/IL-4^hi^/IL-5^hi^/IL-21^lo^) and co-express Bcl6 and GATA3 [242]. This subset is different from Tfh2, cells which are Bcl6/BATF co-expressing IL-4^hi^/IL-21^hi^ Tfh cells. Interestingly, these Tfh13 cells are required for the production of high- but not low-affinity IgE and for subsequent allergen-induced anaphylaxis. Similarly, although class switching occurs infrequently in the germinal centers [221], IFNγ-producing cells with a history of T-bet expression have been found in the germinal centers [61]. Therefore, it is possible that germinal center IFNγ-producing Tfh cells may play an important role in the production of high-affinity IgGs.

It is likely that during activation, naive CD4 T cells may incorporate several layers of fate decisions over time and space at the stages of priming and reinforcement/terminal differentiation [4]. During priming, there are two signals essential for naïve CD4 T cells fate determination: signals downstream of TCR/co-stimulation, which affect non-Tfh versus Tfh cell fate decision, and signals downstream of cytokine receptors, which influence T helper subset decision (e.g., Th1, Th2, or Th17). Therefore, high and low TCR stimulation, which cause a differential pattern of CD25/IRF4/Blimp-1/Bcl6/CXCR5 expression, will result in non-Tfh or Tfh cell fate, respectively. Meanwhile, specific cytokine signals triggered in a specific inflamed environment may lead to the expression of the transcriptional networks that control a specific effector program (e.g., Th1/Th2/Th17 or Tfh1/Tfh2/Tfh17). During reinforcement/terminal differentiation, non-Tfh Teff cells migrate to different tissues depending on the chemokine receptors they express and produce corresponding cytokines, whereas Tfh cells migrate to the T-B border and GC for further maturation and to give help to B cells. As a result, these bifurcated parallel axes allow CD4 T cells to rapidly and effectively augment their particular effector programs in host defense [4]. Since the Tfh and non-Tfh Teff cell differentiation programs may antagonize each other, the fate determination of a particular Tfh cell subset may involve several sequential differentiation steps [223]. Alternatively, the transient expression of non-Tfh Teff cell-associated transcription factors during early Tfh cell differentiation may epigenetically program the fate of a specific subset of Tfh cells [61].

### 6.3. Tfh Related Diseases

Since Tfh cells are critical for the generation of effective long-lived protective antibody responses, the dysregulation of Tfh functions has been noted in various diseases such as inflammation, autoimmunity, allergy, and cancer [32]. Diseases caused by Tfh cells can be divided into two distinct types: autoimmune diseases due to an overabundance of Tfh cells and immunodeficiencies due to a defect in the generation and functions of Tfh cells. One typical representative of autoimmune diseases is systemic lupus erythematosus (SLE), and these patients are found to have increased frequencies of Tfh-like cells in peripheral blood and an amplification of pathogenic autoantibodies [243,244]. Sjogren’s syndrome (SS) [245], juvenile dermatomyositis [246], and rheumatism [247] are other common autoimmune diseases related to Tfh cells. For immunodeficient diseases, mutations of genes, such as *STAT3* [248], *CD40L* [249,250], and *ICOS* [251], which are important for Tfh cell differentiation and functions, were found in immunodeficient patients with reduced numbers of Tfh cells. Given the important role of Tfh cells in helping B cell antibody production, enhancing Tfh cell-associated biology is being considered for effective vaccination, whereas targeting Tfh cells aiming to reduce antibody production could be a base for therapeutic interventions in some human autoimmune diseases.

## 7. Concluding Remarks

In order to protect a host from the invasion of various pathogens, the immune system has evolved a series of mechanisms to respond rapidly and effectively. CD4 T helper subsets mediate effective adaptive immune responses particularly when innate immune responses fail to eliminate pathogens; CD4 T cell responses also generate memory, which can quickly respond to the second infection of the same or similar pathogens. Different types of adaptive immune responses are executed by Th1/Th2/Th17 cells, respectively, with each subset of T helper cells possessing their unique properties in producing their signature cytokines. Interestingly, before adaptive responses take place, innate lymphoid cells, which can also be divided into ILC1/ILC2/ILC3 subsets, may provide early protection and allow time for adaptive responses, whereas memory and/or memory-phenotype CD4 T cells, by possessing some similar features of ILCs (i.e., a completed differentiation process allowing a rapid response, capable of responding to inflammatory cytokines and tissue residency), may behave similarly as ILCs, to a certain extent, during a subsequent infection [252,253].

Th1, Th2, and Th17 cells can be categorized as non-Tfh Teff cells in parallel with Tfh and Treg cells, since both Tfh cells and Treg cells may be further divided into Th1-, Th2-, and Th17-like subpopulations [7,61,183,254]. Cell plasticity may exist between non-Tfh and Tfh cells, and between Teff cells and Treg cells [130,255,256]. However, even within Teff cells, particularly for Th17 cells, cell plasticity has been well documented. Th1-like Th17 cells have been found during infections and in autoimmune settings. The heterogeneity and plasticity of CD4 T cell subsets are largely determined by the temporal and quantitative co-expression of multiple master transcription factors during the differentiation of these cell subsets [7]. Between non-Tfh and Tfh cells, their balance may determine the magnitude of cellular versus humoral responses. In contrast, the balance between Teff and Treg cells reveals the yin and yang sides of the immune system. Understanding such balances is not only important for gaining our knowledge in basic immunology in infections and inflammations, but also for providing great opportunities in treating different types of immunological diseases. In fact, more and more attention is being paid to Treg-based therapies [168], because Treg cells play critical roles in anti-inflammation, autoimmunity prevention, and cancer promotion.

With the rapid development of modern technologies, such as single-cell RNA sequencing [257], CRISPR (clustered regularly interspaced short palindromic repeats)-mediated genome editing [93,258], Cryo-EM (cryogenic electron microscopy) structure analysis [259], single-cell mass spectrometry [260], and immunoimaging [261,262,263], many important findings are being made in the identification, development, and regulation of new CD4 T cell and ILC subsets, as well as their relationship and crosstalks in diseases. In the future, it is also important to investigate how CD4 T cell memory for each subset is formed and maintained [264] and whether the plasticity of antigen cross-reactive CD4 T cell memory cells may shape the primary response to a new infection [265]. With exciting new findings in basic immunology, our better and deeper understanding of this powerful system will help us uncover innovative measures for diseases treatment, thereby improving human health.
